# Associations of food motives with red meat and legume consumption in the population-based DILGOM study

**DOI:** 10.1007/s00394-023-03231-8

**Published:** 2023-08-11

**Authors:** Annukka Hentilä, Satu Männistö, Niina E. Kaartinen, Pekka Jousilahti, Hanna Konttinen

**Affiliations:** 1 University of Helsinki, Helsinki, Finland; 2 Finnish Institute for Health and Welfare, Helsinki, Finland

**Keywords:** Food Choice Questionnaire, Food Frequency Questionnaire, Food choice motives, Legumes, Red meat, Adults

## Abstract

**Purpose:**

To improve human health and environmental sustainability, red meat consumption should decrease and legume consumption increase in diets. More information on food motives, however, is required when developing more tailored and effective interventions targeting legume and meat consumption. We aimed to examine the associations between food motives and red meat and legume consumption, and whether these associations differ between different subgroups (gender, age groups, marital status, education, BMI).

**Methods:**

Ten food motives (health, mood, convenience, sensory appeal, natural content, price-cheap, price-value, weight control, familiarity and ethical concern measured with Food Choice Questionnaire) were studied in 3079 Finnish adults in the population-based DILGOM study. Food consumption was assessed with Food Frequency Questionnaire. The adjusted estimates from multivariable regression models are reported.

**Results:**

Higher relative importance of natural content (β = − 0.275, 95% CI − 0.388; − 0.162) and ethical concern (β = − 0.462, 95% CI − 0.620; − 0.305) were associated with lower red meat consumption, and higher appreciation of sensory appeal (β = 0.482, 95% CI 0.347; 0.616) and price-cheap (β = 0.190, 95% CI 0.099; 0.281) with higher red meat consumption. Higher importance of health (β = 0.608, 95% CI 0.390; 0.825) was associated with higher legume consumption, and higher appreciation of convenience (β = − 0.401, 95% CI − 0.522; − 0.279), price-value (β = − 0.257, 95% CI − 0.380; − 0.133) and familiarity (β = − 0.278, 95% CI − 0.393; − 0.164) with lower legume consumption. The associations of particularly ethical concern, weight control, sensory appeal and mood varied according to gender, age, marital status or BMI.

**Conclusion:**

The development and implementation of actions to decrease red meat and increase legume consumption should focus on several food motives across different subgroups.

**Supplementary Information:**

The online version contains supplementary material available at 10.1007/s00394-023-03231-8.

## Introduction

Climate change and global warming are serious threats to people and environment. The whole food system and especially red meat production is a considerable strain on the environment [1, 2]. Consequently, many positive effects on the environment may be achieved by replacing animal-based protein with plant-based protein, such as legumes, in diets [3]. In addition, high red and processed meat consumption has been associated with many adverse health outcomes [4, 5], whereas legume consumption with positive health outcomes [4, 6]. Sustainable diets have become an important theme in the recently published nutrition recommendations and food-based dietary guidelines, such as the Planetary Health Diet [7], Danish Nutrition Recommendations [8] and upcoming Nordic Nutrition Recommendations 2022 [9]. In the latest Finnish national dietary survey, most adults did not meet the recommendation for fruits and vegetables, and men consumed much more red and processed meat as recommended [10]. In addition, the consumption of legumes was low, only 12–13 g/d [11].

One important set of factors influencing food selection are food motives [12]. Based on the previous literature, taste/sensory appeal, price, convenience and health are the most important food motives for consumers [13–15]. Most previous studies have focused on the absolute importance of food motives. In the present study, we analyzed the relative importance because individuals often have to prioritize motives when making food choices [16]. Even if the absolute importance of food motive would be fairly high, its relative importance can be low, and thus, have only little effect on the decision [17]. In two Finnish studies, health, pleasure/sensory appeal, convenience and price were relatively the most valued food motives [18, 19].

The current evidence on the associations of food motives with red and processed meat and legume consumption is scarce. Only a few studies have investigated specifically the consumption of legumes [20, 21] or red meat [14, 15, 21] and absolute food motives and none have studied processed meat and food motives. In these studies, higher absolute importance of natural content/concerns, health, ethical concern and weight control were associated with lower red meat [14, 20, 21] and higher legume consumption [20, 21], and higher absolute appreciation of convenience with higher red meat [15] and lower legume consumption [21]. In other previous studies, red and processed meat and legume consumption have been a part of either specific dietary pattern (e.g., western dietary pattern) [22] or special diet [19], and therefore it cannot be concluded that food motives were specifically associated with red and processed meat or legumes. Moreover, there is a clear gap of information whether these associations between food motives and red and processed meat and legume consumption vary between different subgroups (e.g., gender, age groups, marital status, education level, BMI). This knowledge is needed to design more tailored and effective interventions to increase the sustainability and healthiness of diets in different population groups.

Our aim was to examine how the relative importance of ten food motives (health, mood, convenience, sensory appeal, natural content, price-cheap, price-value, weight control, familiarity and ethical concern) were associated with red meat and legume consumption in Finnish adults, and whether these associations varied between different subgroups (i.e., gender, age groups, marital status, education level, BMI). In this study, the term “red meat” consisted of red and processed meat.

## Materials and methods

### Participants and setting

**Fig. 1:**
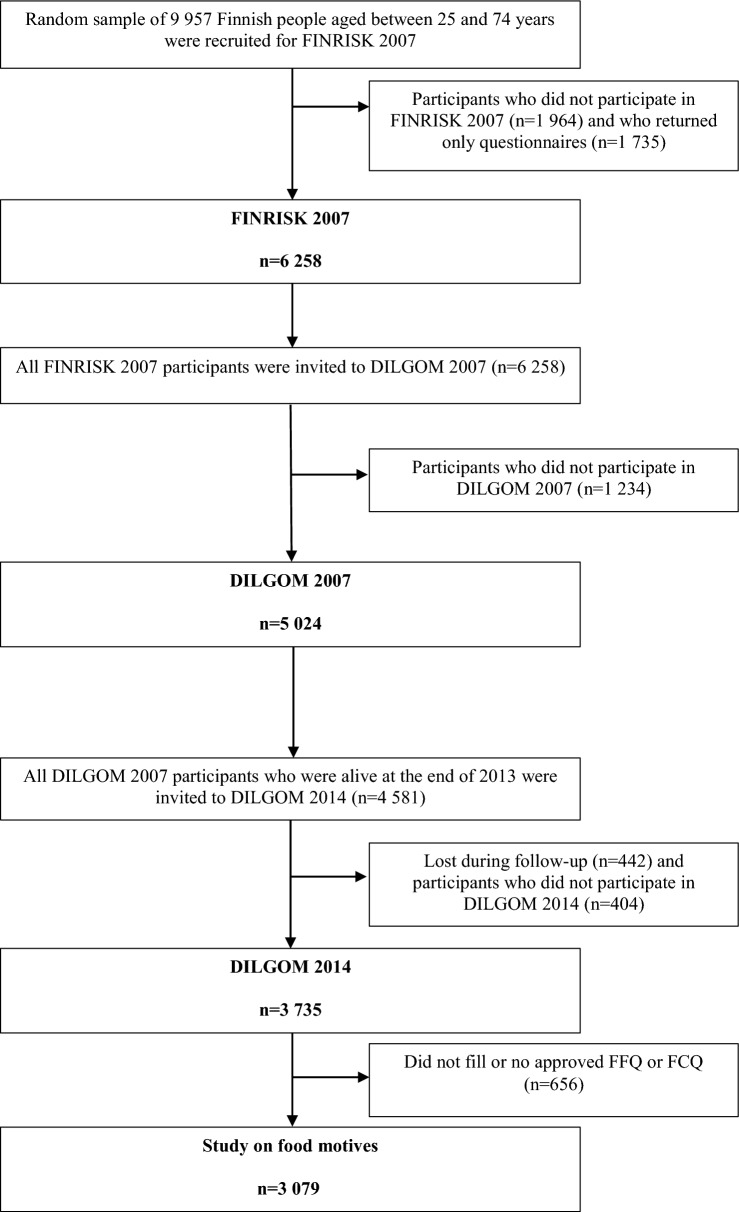
Flowchart of the selection process of study participants

The DIetary, Lifestyle, and Genetic determinants of Obesity and Metabolic syndrome 2007 baseline study (DILGOM 2007) was conducted in 2007 as a part of the FINRISK 2007 Study (FINRISK 2007) and its aim was to examine the role of nutrition, lifestyle, psychosocial factors, environment and genetic factors in the development of obesity and metabolic syndrome [23, 24]. In FINRISK 2007, a random sample of 9 957 Finnish people, aged between 25 and 74 years, was derived from five large study regions: the provinces of North Karelia, North Savo and Oulu, cities of Helsinki and Vantaa, and the areas of Turku and Loimaa. From each region, within each gender and 10-year age group, 200 participants were randomly recruited. All the participants of FINRISK 2007 (n = 6258, between January and March 2007) were invited to participate in DILGOM 2007 between April and June 2007 (Fig. 1). DILGOM 2007 data consisted of 5 024 participants (participation rate 80%). In FINRISK and DILGOM 2007, the participants attended a health examination (including measurements and blood samples) and filled in questionnaires.

Participants who were alive, lived still in Finland and had taken part in DILGOM 2007 (n = 4581) were invited to the DILGOM 2014 follow-up study (DILGOM 2014) between April and June 2014. DILGOM 2014 included 3735 participants (participation rate 82%). DILGOM 2014 was carried out in two ways: the first group of participants from provinces of North Karelia, North Savo and Oulu, received mailed questionnaires and self-reported their weight and height (n = 2423), whereas the other group of participants from the areas of Turku and Loimaa and the cities of Helsinki and Vantaa participated in a health examination where they also completed questionnaires (n = 1312). In this study on food motives, we focused on those 3 288 participants of DILGOM 2014 who had filled in the Food Frequency Questionnaire (FFQ) and Food Choice Questionnaire (FCQ). Of these 3288 participants, 209 were excluded because they had missing values on FCQ or FFQ resulting in the final data of 3079.

### Measures

#### Food consumption

Red meat and legume consumption were assessed using a validated FFQ [25, 26] including 132 food items (related to the Finnish food culture). The participants were asked about their food consumption for previous 12 months. In the FFQ, there were nine options for the consumption frequency for each food item—from “never or seldom” to “more than six times a day.” The portion size of each food item was defined separately by sex (e.g., glass, slice, grams). Both the foods and portion sizes were based on the National FinDiet Survey [27, 28]. The average daily food consumption, nutrients and energy intakes were calculated by the national food composition database (Fineli®) of the Finnish Institute for Health and Welfare. In the present study, the term “red meat” included (both processed and unprocessed) beef, pork, lamb, offal, game, sausages and meat products, and “legumes” consisted of legumes and soy products.

#### Food motives

Food motives were measured using a shortened version of the FCQ [18, 19]. The original FCQ [13] contains thirty-six questions assessing nine motive dimensions, but the factor structure has not been repeated well in other studies done in several countries [29, 30]. Thus, those authors suggested that the FCQ could be developed by reducing the number of food motive dimensions and questions on each dimension [30]. The shortened version of the FCQ used in this study included 25 items from the original FCQ (at least two items per dimension) and three additional items tapping ethical aspects of food selection [19]. For each item, the participants were asked to evaluate how much they agreed with the statement ‘‘It is important to me that the food I eat on a typical day...’’ using a four-point scale (1 = not at all important, 2 = a little important, 3 = moderately important, 4 = very important). In line with the factor analytic results of the previous Finnish study [19], we examined ten food motives in this study: health, mood, convenience, sensory appeal, natural content, price-cheap, price-value, weight control, familiarity and ethical concern. The two price items ("is cheap" reflecting more the influence of monetary resources, and "is good value for money" reflecting more the concept of worth) were analyzed as separate motives because they correlated relatively weakly with each other both in the previous study (r = 0.22) [19] and in the present sample (r = 0.23).

The relative importance of each food motive was calculated similarly to two previous Finnish studies [18, 19]: first, a mean score across the items measuring the respective motive was calculated (except for price-cheap and price-value which were analyzed as single items). Second, this mean score (or the score for price-cheap or price-value item) was divided by the respondent’s mean score across all 28 motive items. For these resulting variables, the scores more than 1 meant that the food motive was rated higher compared to the mean and less than 1 the opposite. Internal reliability was mostly good and at least acceptable for each food motive dimension measured using two or more items: health (Cronbach’s α = 0.83), mood (α = 0.65), convenience (α = 0.69), sensory appeal (α = 0.72), natural content (α = 0.79), weight control (α = 0.80), familiarity (α = 0.78) and ethical concern (α = 0.82).

#### Background variables

Sociodemographic variables included gender (men, women), age in years, marital status and education level. Information on marital status was derived using a question with six options: “married”, “cohabiting”, “unmarried”, “judicial separation or divorced”, “widowed” and “registered partnership.” In this study, marital status was categorized into two categories: “married/cohabiting” and “other.” Education level was assessed via self-reported total years of education, and they were divided into tertiles (representing low, middle and high education) according to birth year.

Participant’s weight and height were either measured in the health examination (two study regions) or they were self-reported (three study regions) in the questionnaire. BMI (kg/m^2^) was calculated, and it was categorized as “participants with obesity (BMI ≥ 30 kg/m^2^)” and “participants without obesity (BMI < 30 kg/m^2^).” In DILGOM, it has previously been shown that self-reported anthropometrics are well in accordance with professionally-measured values [31].

### Statistical methods

The statistical analyses were done with IBM SPSS Statistics, version 27. The differences between genders and age, education, marital status and BMI groups regarding red meat and legume consumption and relative food motives were studied using One-Way ANOVA (in the case that Levene’s test indicated equal variances across groups) or Welch test (in the case that Levene’s test indicated unequal variances). Two linear regression models were used to test the associations between food motives (predictor variables) and red meat and legume consumption (outcome variables). The first model was un-adjusted model and the second was multivariable model with adjusting variables (age, gender, education, marital status, BMI and energy intake) which were selected based on the previous literature. Regression models were all done separately for each food motive and food consumption variable. Because red meat and legume consumption were not normally distributed, they were altered to their natural logarithms. In One-Way ANOVA and Welch tests, Partial Eta Squared was used to evaluate effect size (i.e. the magnitude of the associations), while standardized regression coefficients were used in linear regression models. The effect size was judged against criteria proposed by Cohen for Partial Eta Squared: very small (< 0.01), small (0.01 to < 0.06), moderate (0.06 to < 0.14), and large (≥ 0.14), and for standardized regression coefficients: very small (< 0.10), small (0.10 to < 0.30), moderate (0.30 to < 0.50), and large (≥ 0.50) [32]. The interactions between different subgroups (i.e., gender, age groups, marital status, education level, BMI) and food motives were studied by linear regression analyses, and when the interaction was statistically significant analyses stratified by the respective subgroups were done. p values and 95% confidence intervals were given for unstandardized regression coefficients. p values < 0.05 were considered statistically significant.

## Results

As shown in Table 1, slightly higher percentage of participants were women (54%). Participants were on average 60 years old (SD 12.9), average education was 13 years (SD 3.9) and a large proportion was married/cohabiting (74%). Mean BMI was 26.8 kg/m^2^ (SD 4.6) and participants with obesity (BMI ≥ 30 kg/m^2^) represented a fifth of the sample.

**Table 1:** Descriptive characteristics of study participants (*n* = 3079)

	Value
Women, %	54.3
Age (y), mean (SD)	59.5 (12.9)
Age groups^a^, %	
31–53	33.5
54–66	33.6
67–82	32.9
Education (y), mean (SD)	12.9 (3.9)
Education groups^b^, %	
Low education	26.5
Middle education	34.8
High education	38.7
Married/cohabiting, %	73.5
BMI (kg/m^2^), mean (SD)	26.8 (4.6)
Participants with obesity (BMI ≥ 30), %	19.9

The proportion of participants with missing data for marital status, education and BMI variables varied from 0.2 to 1.1%

^a^Tertiles

^b^Education years divided into tertiles according to birth year

Men consumed more red meat (includes also processed meat) than women (p < 0.001) and the difference between genders was moderate in size (Partial Eta^2^ = 0.099) (Table 2). Mean consumption of red meat was 165 g/d (SD = 104.2) (19.7 g/MJ) for men and 104 g/d (SD = 78.3) (12.4 g/MJ) for women. Between age groups, there were no statistically significant difference in red meat consumption. In the youngest age group, the red meat consumption was 137 g/d (SD = 95.9) (16.4 g/MJ), in the middle group 130 g/d (SD = 91.4) (15.5 g/MJ) and in the oldest group 129 g/d (SD = 100.2) (15.4 g/MJ). Married/cohabiting participants consumed more red meat than other participants (p = 0.000) and the difference was small in size (Partial Eta^2^ = 0.016) (Supplementary Table 1). Mean consumption of red meat was 137 g/d (SD = 96.2) (16.4 g/MJ) for married/cohabiting participants and 117 g/d (SD = 93.9) (14.0 g/MJ) for other participants. Participants with the lowest education consumed more red meat than participants with higher education (p = 0.000) but the difference was very small in size (Partial Eta^2^ = 0.006). Mean consumption of red meat was 143 g/d (SD = 111.8) (17.1 g/MJ) for participants with the lowest education, 134 g/d (SD = 95.4) (16.0 g/MJ) for those with the middle education and 123 g/d (SD = 83.4) (14.7 g/MJ) for those with the highest education. Participants with obesity consumed more red meat than those without obesity (p < 0.001) but the difference was very small in size (Partial Eta^2^ = 0.004) (Supplementary Table 2). Mean consumption of red meat was 128 g/d (SD = 92.5) (15.3. g/MJ) for participants without obesity and 145 g/d (SD = 108.1) (17.3 g/MJ) for participants with obesity. Men consumed legumes more than women (p = 0.015) but the size of the difference between genders was very small (Partial Eta^2^ = 0.002). Mean consumption of legumes was 17 g/d (SD = 16.8) (2.0 g/MJ) for men and 15 g/d (SD = 16.7) (1.8 g/MJ) for women. The oldest age group (67–82 y) consumed the most legumes compared to other age groups (p < 0.007) but the difference between groups was very small in size (Partial Eta^2^ = 0.003). The oldest age group consumed legumes on average 17 g/d (SD = 19.6) (2.0 g/MJ) and the younger age groups 15–16 g/d (SD = 14.7, SD = 15.7) (1.8 g/MJ and 1.9 g/MJ). There was no statistically significant difference between married/cohabiting participants’ and other participants’ legume consumption. Mean legume consumption was 16 g/day (SD = 16.6) (1.9 g/MJ) for married/cohabiting participants and 16 g/d (SD = 17.4) (1.9 g/MJ) for other participants. No statistically significant difference between education groups was observed in legume consumption. Mean legume consumption was 16 g/d (SD = 18.9) (1.9 g/MJ) for participants with the lowest education, 15 g/d (SD = 14.1) (1.8 g/MJ) for those with the middle education and 17 g/d (SD = 17.5) (2.0 g/MJ) for those with the highest education. There was no statistically significant difference between participants with and without obesity in legume consumption. Mean legume consumption was 16 g/d (SD = 16.1) (1.9 g/MJ) for participants without obesity and 17 g/d (SD = 19.6) (2.0 g/MJ) for participants with obesity.

**Table 2:** Mean consumption of red meat and legumes and relative food motive mean scores by gender and different age groups

	Women		Men		p^a^	Partial Eta^2,b^	31–53 y		54–66 y		67–82 y		p^a^	Partial Eta^2,b^
	Mean	SD	Mean	SD			Mean	SD	Mean	SD	Mean	SD		
Red meat consumption^c^ (g/d)	104	78.3	165	104.2	< 0.001^f^	0.099	137	95.9	130	91.4	129	100.2	0.127^g^	0.001
Legume consumption^d^ (g/d)	15	16.7	17	16.8	0.015^g^	0.002	15	14.7	16	15.7	17	19.6	0.007^f^	0.003
Energy intake (kJ)	8862	3154	10,867	3920	0.000^g^	0.074	9521	3484	9766	3587	10 051	3894	0.005^g^	0.003
Food motive^e^														
Health	1.08	0.11	1.07	0.13	0.002^f^	0.003	1.06	0.13	1.07	0.11	1.08	0.11	< 0.001^f^	0.006
Mood	0.98	0.15	0.97	0.16	0.071^f^	0.001	1.00	0.16	0.98	0.15	0.96	0.15	< 0.001^g^	0.013
Convenience	0.99	0.21	0.98	0.21	0.321^g^	0.000	1.06	0.22	0.97	0.21	0.94	0.18	< 0.001^f^	0.058
Sensory appeal	1.12	0.16	1.13	0.18	0.167^f^	0.001	1.18	0.19	1.11	0.17	1.08	0.15	< 0.001^f^	0.054
Natural content	1.02	0.20	1.00	0.21	< 0.001^f^	0.004	0.97	0.23	1.03	0.20	1.04	0.18	< 0.001^f^	0.017
Price-cheap	1.01	0.24	1.03	0.26	0.010^f^	0.002	1.02	0.27	1.02	0.25	1.01	0.23	0.557^f^	0.000
Price-value	1.16	0.19	1.22	0.22	< 0.001^f^	0.022	1.22	0.23	1.18	0.20	1.16	0.19	< 0.001^f^	0.017
Weight control	0.98	0.17	0.94	0.19	< 0.001^f^	0.007	0.94	0.19	0.98	0.18	0.96	0.17	< 0.001^f^	0.010
Familiarity	0.84	0.23	0.92	0.23	< 0.001^g^	0.030	0.85	0.25	0.87	0.22	0.92	0.21	< 0.001^f^	0.014
Ethical concern	0.90	0.14	0.89	0.16	0.608^f^	0.000	0.85	0.16	0.90	0.14	0.94	0.13	< 0.001^f^	0.058

^a^ANOVA (equal variances, Levene’s test p > 0.05) or Welch test (unequal variances, Levene’s test p < 0.05) was used to test differences between genders and age groups

^b^Effect size was judged against criteria proposed by Cohen [32] for Partial Eta Squared: very small (< 0.01), small (0.01 to < 0.06), moderate (0.06 to < 0.14), and large (≥ 0.14)

^c^Beef, pork, lamb, game, offal, sausage and meat products

^d^Bean, peas and soy products

^e^Scores > 1 for each relative food motive reflect that it was rated more important compared to the mean and < 1 the opposite

^f^Welch test

^g^ANOVA

Of the food motives, the highest relative importance was for price-value, sensory appeal and health and the lowest for weight control, ethical concern and familiarity (Table 2). In general, there were many statistically significant differences between men and women, but the most prominent differences were for price-value (Partial Eta^2^ = 0.022) and for familiarity (Partial Eta^2^ = 0.030) even though the effect sizes were small. Men appreciated more price-value and familiarity than women. Many statistically significant differences were also observed between age groups, but the most notable differences (small to moderate in size) were for convenience (Partial Eta^2^ = 0.058), ethical concern (Partial Eta^2^ = 0.058) and sensory appeal (Partial Eta^2^ = 0.054). Convenience and sensory appeal were the most important to the youngest age group (31–53 y) and ethical concern to the oldest age group (67–82 y).

In the multivariable linear regression models, higher importance of health (std. β = − 0.052, p < 0.001), natural content (std. β = − 0.071, p < 0.001) and ethical concern (std. β = − 0.088, p < 0.001) were associated with lower consumption of red meat (Table 3). In contrast, higher importance of mood (std. β = 0.039, p = 0.009), convenience (std. β = 0.042, p = 0.006), sensory appeal (std. β = 0.106, p < 0.001), price-cheap (std. β = 0.061, p < 0.001) and price-value (std. β = 0.035, p = 0.020) were associated with higher red meat consumption. The size of the association between food motives and red meat consumption was the most prominent, but small, for sensory appeal, natural content, price-cheap and ethical concern.

**Table 3:** Associations between relative food motives and red meat and legume consumption in linear regression models

	Red meat consumption^a^				Legume consumption^b^			
	β^c^	95% CI for β^e^	std. β^d^	p^e^	β^c^	95% CI for β^e^	std. β^d^	p^e^
Health								
Model 1	− 0.601	− 0.835; − 0.366	− 0.090	< 0.001	0.564	0.344; 0.793	0.087	< 0.001
Model 2	− 0.347	− 0.545; − 0.150	− 0.052	< 0.001	0.608	0.390; 0.825	0.093	< 0.001
Mood								
Model 1	0.375	0.196; 0.554	0.074	< 0.001	0.209	0.034; 0.384	0.042	0.019
Model 2	0.198	0.049; 0.347	0.039	0.009	0.087	− 0.078; 0.252	0.017	0.302
Convenience								
Model 1	0.118	− 0.013; 0.248	0.032	0.077	− 0.433	− 0.559; − 0.307	− 0.120	< 0.001
Model 2	0.156	0.045; 0.266	0.042	0.006	− 0.401	− 0.522; − 0.279	− 0.112	< 0.001
Sensory appeal								
Model 1	0.487	0.328; 0.647	0.107	< 0.001	− 0.196	− 0.352; − 0.040	− 0.044	0.014
Model 2	0.482	0.347; 0.616	0.106	< 0.001	− 0.141	− 0.290; 0.009	− 0.032	0.065
Natural content								
Model 1	− 0.412	− 0.547; − 0,277	− 0.107	< 0.001	0.145	0.012; 0.277	0.039	0.032
Model 2	− 0.275	− 0.388; − 0.162	− 0.071	< 0.001	0.181	0.056; 0.307	0.048	0.005
Price-cheap								
Model 1	0.264	0.155; 0.374	0.085	< 0.001	− 0.076	− 0.184; 0.031	− 0.025	0.162
Model 2	0.190	0.099; 0.281	0.061	< 0.001	− 0.087	− 0.188; 0.014	− 0.029	0.090
Price-value								
Model 1	0.268	0.135; 0.400	0.071	< 0.001	− 0.275	− 0.405; − 0.145	− 0.075	< 0.001
Model 2	0.132	0.021; 0.244	0.035	0.020	− 0.257	− 0.380; − 0.133	− 0.070	< 0.001
Weight control								
Model 1	− 0.281	− 0.435; − 0.127	− 0.064	< 0.001	0.075	− 0.076; 0.226	0.018	0.328
Model 2	− 0.064	− 0.193; 0.065	− 0.015	0.329	0.167	0.024; 0.309	0.039	0.022
Familiarity								
Model 1	0.353	0.233; 0.473	0.103	< 0.001	− 0.185	− 0.302; − 0.067	− 0.055	0.002
Model 2	0.080	− 0.024; 0.184	0.023	0.134	− 0.278	− 0.393; − 0.164	− 0.084	< 0.001
Ethical concern								
Model 1	− 0.444	− 0,629; − 0.258	− 0.084	< 0.001	0.355	0.173; 0.536	0.069	< 0.001
Model 2	− 0.462	− 0.620; − 0.305	− 0.088	< 0.001	0.278	0.103; 0.452	0.054	0.002

Model 1 = crude model

Model 2 = multivariable model adjusted for age (continuous), gender (dichotomous), education (continuous), marital status (dichotomous), BMI (continuous) and energy intake (continuous)

^a^Beef, pork, lamb, offal, game, sausages and meat products

^b^Bean, peas and soy products

^c^Unstandardized regression coefficients

^d^Standardized regression coefficients. Effect size was judged against criteria proposed by Cohen [32] for standardized coefficients: very small (< 0.10), small (0.10 to < 0.30), moderate (0.30 to < 0.50), and large (≥ 0.50)

^e^p values and 95% confidence intervals were given for unstandardized regression coefficients

In the multivariable models, higher importance of health (std. β = 0.093, p < 0.001), natural content (std. β = 0.048, p = 0.005), weight control (std. β = 0.039, p = 0.022) and ethical concern (std. β = 0.054, p = 0.002) were associated with higher legume consumption. Conversely, higher importance of convenience (std. β = − 0.112, p < 0.001), price-value (std. β = − 0.070, p < 0.001) and familiarity (std. β = − 0.084, p < 0.001) were associated with lower consumption of legumes (Table 3). The size of the association between food motives and legume consumption was the most notable, but small, for health, convenience, price-value and familiarity.

We observed some differences between subgroups (i.e., gender, age groups, marital status, BMI) in the associations of food motives with red meat and legume consumption (Table 4). In women, higher importance of ethical concern was associated with higher legume consumption, but not in men. In the younger age groups (31–53 y, 54–66 y), higher importance of ethical concern was associated with lower red meat consumption and higher legume consumption, while in the oldest age group (67–82 y) the association was not significant. In the oldest age group, higher importance of weight control was associated with higher legume consumption, but in the younger age groups there were no significant associations. In participants who were married/cohabiting, higher appreciation of sensory appeal and lower appreciation of ethical concern were associated with higher red meat consumption. The results were similar in participants who were unmarried or did not live with a partner, but the associations were stronger. In those who were unmarried or did not live with a partner, higher appreciation of mood was associated with higher legume consumption, but not in married/cohabiting participants. In participants with obesity, lower red meat consumption was associated with higher importance of weight control, higher red meat consumption with greater importance of mood and higher legume consumption with higher appreciation of ethical concern. All other interactions for BMI, marital status, age groups and gender were statistically non-significant, and none of the interactions for education level was significant.

**Table 4:** Associations between relative food motives (predictors) and red meat and legume consumption (outcomes) by gender, age groups, marital status and BMI groups

	Red meat consumption^a^			Legume consumption^b^		
	β^c^	95% CI for β	p	β	95% CI for β	p
Weight control						
Age groups						
31–53 y	−	−	−	0.003	− 0.213; 0.220	0.975
54–66 y	−	−	−	0.174	− 0.077; 0.426	0.174
67–82 y	−	−	−	0.398	0.116; 0.680	0.006
Interaction terms^d^						
31–53 y	0.220	− 0.094; 0.535	0.169	− 0.445	− 0.792;− 0.098	0.012
54–66 y	0.097	− 0.231; 0.426	0.560	− 0.246	− 0.608; 0.116	0.184
67–82 y	Ref			Ref		
BMI groups						
BMI < 30 (kg/m^2^)	0.044	− 0.097; 0.185	0.545	−	−	−
BMI ≥ 30 (kg/m^2^)	− 0.355	− 0.676; − 0.035	0.030	−	−	−
Interaction term^d^	− 0.420	− 0.766; − 0.073	0.018	0.143	− 0.238; 0.524	0.462
Ethical concern						
Gender						
Women	−	−	−	0.472	0.224; 0.719	< 0.001
Men	−	−	−	0.105	− 0.142; 0.351	0.405
Interaction term^d^	− 0.121	− 0.425; 0.182	0.433	0.404	0.067; 0.740	0.019
Age groups						
31–53 y	− 0.745	− 0.993;− 0.498	< 0.001	0.469	0.214; 0.724	< 0.001
54–66 y	− 0.351	− 0.625;− 0.077	0.012	0.303	0.002; 0.603	0.048
67–82 y	− 0.134	− 0.440; 0.173	0.392	− 0.110	− 0.479; 0.259	0.558
Interaction terms^d^						
31–53 y	− 0.586	− 0.983;− 0.190	0.004	0.624	0.184; 1.064	0.005
54–66 y	− 0.245	− 0.659; 0.169	0.247	0.388	− 0.071; 0.848	0.098
67–82 y	Ref			Ref		
Marital status						
Married/cohabiting	− 0.320	− 0.494;− 0.146	< 0.001	−	−	−
Others	− 0.805	− 1.146;− 0.465	< 0.001	−	−	−
Interaction term^d^	− 0.421	− 0.762;− 0.080	0.016	− 0.134	− 0.512; 0.245	0.489
BMI groups						
BMI < 30 (kg/m^2^)	−	−	−	0.180	− 0.013; 0.374	0.067
BMI ≥ 30 (kg/m^2^)	−	−	−	0.631	0.229; 1.034	0.002
Interaction term^d^	0.018	− 0.370; 0.406	0.926	0.556	0.127; 0.984	0.011
Sensory appeal						
Marital status						
Married/cohabiting	0.384	0.236; 0.532	< 0.001	−	−	−
Others	0.776	0.479; 1.073	< 0.001	−	−	−
Interaction term^d^	0.302	0.005; 0.599	0.046	0.155	− 0.176; 0.485	0.359
Mood						
Marital status						
Married/cohabiting	−	−	−	− 0.044	− 0.230; 0.143	0.645
Others	−	−	−	0.456	0.110; 0.803	0.010
Interaction term^d^	− 0.239	− 0.575; 0.097	0.163	0.485	0.114; 0.857	0.010
BMI groups						
BMI < 30 (kg/m^2^)	0.133	− 0.035; 0.300	0.120	−	−	−
BMI ≥ 30 (kg/m^2^)	0.531	0.192; 0.869	0.002	−	−	−
Interaction term^d^	0.419	0.045; 0.794	0.028	− 0.289	− 0.702; 0.124	0.170
Familiarity						
Marital status						
Married/cohabiting	0.035	− 0.079; 0.149	0.543	−	−	−
Others	0.168	− 0.066; 0.402	0.158	−	−	−
Interaction term^d^	0.256	0.032; 0.479	0.025	− 0.139	− 0.385; 0.108	0.270

Gender, age group, marital status and BMI group stratified results are reported only for those associations with the corresponding interaction term p < 0.05

Stratified models are adjusted for age, gender, education, marital status, BMI (except the respective stratifying factor) and energy intake

^a^Beef, pork, lamb, game, offal, sausages and meat products

^b^Bean, peas and soy products

^c^Unstandardized regression coefficients

^d^Models testing the interactions include the respective food motive variable, age, gender, education, marital status, BMI, energy intake, and the interaction term between the food motive and sociodemographic factor or BMI

## Discussion

The present study added knowledge on the associations between the relative importance of food motives, red meat (including also processed meat) and legume consumption across different subgroups (i.e., gender, age groups, marital status, education level, BMI). We found that participants who considered health, natural content and ethical concern more important consumed less red meat (Table 5). In contrast, participants who valued more mood, convenience, sensory appeal, price-cheap and price-value consumed more red meat. Previous studies have also associated lower red meat consumption with higher importance of health [14, 22], natural content and ethical concern [14], as well as with lower importance of convenience [15, 20] and price [20]. Regarding legume consumption, we found that those who valued more health, natural content, weight control and ethical concern consumed more legumes. On the contrary, participants who considered convenience, price-value and familiarity more important consumed less legumes. There are similar findings in earlier studies; higher importance of natural concerns, health and weight control were related to higher legume consumption and higher importance of convenience [20] and price [20, 22] were associated with lower legume consumption.

**Table 5:** Summary of the associations between food consumption and food motives in the present study^a^

	Higher red meat consumption^b^	Higher legume consumption^c^
Health	–	+ *
Mood	+	ns
Convenience	+	–*
Sensory appeal	+ *	ns
Natural content	–*	+
Price-cheap	+ *	ns
Price-value	+	–*
Weight control	ns	+
Familiarity	ns	–*
Ethical concern	–*	+

^a^Statistically significant positive association (+), statistically significant inverse association (−), non-significant association (ns), most notable associations based on effect size (*)

^b^Beef, pork, lamb, offal, game, sausages and meat products

^c^Bean, peas and soy products

The most notable associations (albeit still small) regarding red meat consumption when effect sizes were considered in our study, were for sensory appeal, natural content, price-cheap and ethical concern. Enjoying eating meat and not wanting to change food habits [33] and disliking the taste of legumes [34] are barriers for decreasing red meat consumption. This could explain why people who appreciated sensory appeal more had also higher red meat consumption in our study. Furthermore, it has been found that people tend to like the taste of foods that are familiar to them [35]. Red meat is a staple food in Finnish diets whereas apart from green peas and pea soup legumes are not a major part of the Finnish food culture [10, 11]. A previous American study found that about 40% of participants considered “clean” (e.g., eating foods without food additives) eating as healthy [36]. This “clean” eating is related to the natural content motive of the FCQ, which has one question on food additives. Perhaps people who value more natural content consume less meat because especially processed meat can contain food additives. Participants with higher red meat consumption considered cheapness of food more important and further analyses revealed that the association concerned particularly processed meat. This could be explained by the fact that food cheapness is more important for people with lower socioeconomic position [19] and in Finland, especially, people with low socioeconomic position consume more processed meat [10]. Also, it was found that people who think that they have enough money compared to their necessities, have lower red and processed meat consumption [37].

When effect sizes were considered, the most notable associations (albeit still small) regarding legume consumption were for health, convenience, price-value and familiarity. In a Mexican study, most of the participants considered legumes as healthy [38]. Higher importance of health when legume consumption was higher might be linked to the perception of legumes as healthy. Lower importance of convenience and familiarity when legume consumption was higher could be related to experiences that preparing legumes takes more time than meat products [34] and the recipes are not that familiar.

Most of the subgroup differences observed in the associations concerned ethical concern motive with the findings complementing the respective previous research conducted in the overall adult population [14, 15]. In those who were not married or did not live with a partner, lower appreciation of ethical concern and higher appreciation of sensory appeal was associated with higher red meat consumption more strongly compared to those who were married or cohabiting. People who live alone can more easily follow their own motivation in food choices, whereas people who live with others must also consider what others prefer. In women, higher importance of ethical concern was associated with higher legume consumption, but not in men. Furthermore, higher importance of ethical concern was associated with lower red meat and higher legume consumption in the two youngest age groups, but not in the oldest one. One explanation could be that younger age groups and women have a better knowledge on the impact of red meat and legume consumption on environmental problems and climate change. Our findings yet suggest that actions aiming to increase legume consumption and decrease red meat consumption in older adults and men should probably focus more on other aspects than the ethicality of diet. In married and cohabiting individuals, strategies that take into account each household member’s food motives (and potential motivational differences between the members) could be effective. Finally, we noted that some food motives (i.e., weight control, mood and ethical concern) were related to red meat or legume consumption particularly in individuals with obesity potentially reflecting the interplay between motivational factors, weight gain and weight control attempts in the current food-rich environments.

The main strength of this study was the large population-based sample of Finnish adults. The validated methods [13, 25, 26] of food consumption and food motives were also used. It has been shown that the FCQ is a valid method to assess food motives in different countries [39]. Furthermore, we have shown earlier (DILGOM 2007) that food consumption was more strongly associated with the relative food motives than the absolute motives [18].

Several limitations of this study need to be acknowledged. Even though the original sample was randomly selected from the Finnish population, there were non-participants through the studies and years. Individuals who participated in DILGOM 2014 were more likely to be older, women, have lower BMI and waist circumference and higher education than those who attended only DILGOM 2007 [40]. Compared to the Finnish Population Data, the participants were more likely women and older, but their education level was similar [41, 42]. The non-participation affects the generalizability of our results. It is likely that the participants of the present health-related study are more interested in their food consumption and health. The results could have been more prominent especially for the red meat consumption with more even gender distribution. One limitation that must be considered is that the data was collected in 2014. It is possible that food motives and related eating habits have changed during this time and especially during the COVID-19 pandemic [43]. A few studies, however, suggest that the changes in food motives have been temporary and long-term effects cannot be determined yet [44, 45]. In a Finnish study, most people reported that the pandemic did not change their eating habits [46]. Another Finnish study conducted in 2018 reported similar observations on the relative importance of different food motives than in the present study using the same version of the FCQ [19]. Therefore, we assume that the current findings could still be fairly relevant after the COVID-19 pandemic because it seems that at least permanent changes in food motives do not occur that quickly. The FFQ tends to overestimate food consumption compared to food records [47], however, the FFQ is the primary method for large epidemiological studies. There can also be memory biases because the FFQ was filled retrospectively. Furthermore, people tend to overestimate their healthy food choices [25], thus, the reported legume consumption could be higher than the actual consumption. The FCQ aims to assess motives that are associated with daily food choices, but it might not include all currently relevant motives, such as the importance of sustainability [13]. Therefore, it could be useful to update some of the motive dimensions in the FCQ. We must also consider that even though individuals might appreciate certain food motives, these motives are not always reflected in their actual behaviour (representing the well-known attitude-behaviour gap). One further limitation is that the cross-sectional study design does not allow conclusions to be drawn on causality. However, as motivational factors are one important set of determinants of daily behaviour, it is reasonable to assume that food motives influence red meat and legume consumption.

This study provides new information on the links between various food motives and red meat and legume consumption. There are very few earlier studies concentrating on processed meat as well. An additional novel aspect of our study is that we identified certain differences between genders and age, marital status and BMI groups in these associations. We thus gained more elaborated knowledge, which can be useful for food industry, nutrition professionals and policy makers. Because taste was more important for those who consumed more red meat, it brings challenges for food manufacturers to produce tasty plant-based food for them. Moreover, familiarity was more important for those who consumed less legumes, thus, it would probably be useful to increase the familiarity of legume- and other plant-based products. It should though be noted that after the present study data from 2014, many plant-based products have been introduced to the market, and especially plant-based protein products have tried to mimic meat products as much as possible. The cheapness of food was more important for those with higher red meat consumption, which highlights the relevance of policy measures to increase the affordability of plant-based food products.

Lunch or work canteens potentially have an important role in increasing the familiarity and consumption of legumes [47]. With the help of nutrition professionals, menus could have more plant-based products. For instance, part of the meat in familiar dishes could be replaced with legumes. Nutrition professionals should also focus on teaching people how to prepare legumes because inability to cook legumes and lack of knowledge on suitable recipes are common obstacles for legume consumption [48, 49]. These kinds of strategies can eventually lead to more sustainable and healthy diets by enhancing the familiarity of legumes and people’s competence to prepare them.

## Conclusion

In summary, the present findings suggest that people with higher red meat consumption are more appreciative of sensory appeal and cheapness of food and less appreciative of natural content and ethical concern. Moreover, people with higher legume consumption place more importance on health and less importance on convenience, familiarity and price-value considerations. We further found that the associations of some food motives (particularly ethical concern, weight control, sensory appeal and mood) varied according to gender, age groups, marital status or BMI. Knowing the most important food motives regarding red meat and legume consumption across population groups may increase the possibility of altering individuals’ food consumption to healthier and more sustainable direction.

### Supplementary Information

Below is the link to the electronic supplementary material.

Supplementary file1 (DOCX 59 KB)

## Data Availability

The DILGOM data are included in the THL Biobank (https://thl.fi/en/web/thl-biobank). The data used in the present study can be made available on request to the THL Biobank according to the given ethical guidelines and Finnish legislation.
