# Supplementary material for: Associations of food motives with red meat and legume consumption in the population-based DILGOM study
Source: Eur J Nutr. 2023 Aug 11;62(8):3263–75. doi: 10.1007/s00394-023-03231-8 (PMC10611614; doi:10.1007/s00394-023-03231-8)
Supplement: Supplementary file 1 — Supplementary file1 (DOCX 59 KB) [file 394_2023_3231_MOESM1_ESM.docx]

**Supplementary Information**

**Article title:** Associations of food motives with red meat and legume consumption in the population-based DILGOM study

**Journal:** European Journal of Nutrition

**Authors:** Annukka Hentilä, Satu Männistö, Niina E. Kaartinen, Pekka Jousilahti, Hanna Konttinen

**Corresponding author:** Hanna Konttinen, Faculty of Social Sciences, University of Helsinki, Helsinki, Finland. E-mail: hanna.konttinen@helsinki.fi

**Supplementary Table 1**. Mean consumption of red meat and legumes and relative food motive mean scores by marital status and education.

|  | Married/Cohabiting | |  | Others | |  | P^a^ | Partial Eta^2,b^ |  | Low education | |  | Middle education | |  | High education | |  | P^a^ | Partial Eta^2,b^ |
| --- | --- | --- | --- | --- | --- | --- | --- | --- | --- | --- | --- | --- | --- | --- | --- | --- | --- | --- | --- | --- |
|  | Mean | SD |  | Mean | SD |  |  |  |  | Mean | SD |  | Mean | SD |  | Mean | SD |  |  |  |
| Red meat consumption^c^ (g/d) | 137 | 96.2 |  | 117 | 93.9 |  | 0.000^f^ | 0.016 |  | 143 | 111.8 |  | 134 | 95.4 |  | 123 | 83.4 |  | 0.000^g^ | 0.006 |
| Legume consumption^d^ (g/d) | 16 | 16.6 |  | 16 | 17.4 |  | 0.094^f^ | 0.001 |  | 16 | 18.9 |  | 15 | 14.1 |  | 17 | 17.5 |  | 0.077^g^ | 0.002 |
| Energy intake (kJ) | 10 003 | 3671 |  | 9167 | 3574 |  | 0.000^g^ | 0.010 |  | 9826 | 3985 |  | 9736 | 3724 |  | 9784 | 3378 |  | 0.868^f^ | 0.000 |
| Food motive^e^ |  | |  |  | |  |  |  |  |  | |  |  | |  |  | |  |  |  |
| Health | 1.07 | 0.12 |  | 1.08 | 0.12 |  | 0.707^g^ | 0.000 |  | 1.06 | 0.11 |  | 1.07 | 0.12 |  | 1.08 | 0.13 |  | 0.000^f^ | 0.007 |
| Mood | 0.98 | 0.16 |  | 0.98 | 0.15 |  | 0.912^g^ | 0.000 |  | 0.98 | 0.15 |  | 0.98 | 0.15 |  | 0.98 | 0.16 |  | 0.599^f^ | 0.000 |
| Convenience | 0.98 | 0.21 |  | 1.02 | 0.23 |  | 0.000^g^ | 0.009 |  | 0.99 | 0.20 |  | 0.99 | 0.22 |  | 0.99 | 0.22 |  | 0.899^f^ | 0.000 |
| Sensory appeal | 1.13 | 0.17 |  | 1.11 | 0.17 |  | 0.005^g^ | 0.003 |  | 1.13 | 0.16 |  | 1.13 | 0.17 |  | 1.13 | 0.19 |  | 0.951^f^ | 0.000 |
| Natural Content | 1.02 | 0.20 |  | 1.00 | 0.22 |  | 0.109^f^ | 0.001 |  | 0.99 | 0.19 |  | 1.01 | 0.20 |  | 1.02 | 0.21 |  | 0.003^g^ | 0.004 |
| Price-cheap | 1.01 | 0.24 |  | 1.05 | 0.27 |  | 0.000^f^ | 0.007 |  | 1.07 | 0.23 |  | 1.02 | 0.25 |  | 0.98 | 0.26 |  | 0.000^f^ | 0.016 |
| Price-value | 1.19 | 0.21 |  | 1.18 | 0.21 |  | 0.308^g^ | 0.000 |  | 1.18 | 0.20 |  | 1.18 | 0.20 |  | 1.19 | 0.22 |  | 0.180^g^ | 0.001 |
| Weight control | 0.97 | 0.18 |  | 0.95 | 0.19 |  | 0.007^f^ | 0.003 |  | 0.95 | 0.17 |  | 0.96 | 0.18 |  | 0.97 | 0.19 |  | 0.138^f^ | 0.001 |
| Familiarity | 0.88 | 0.23 |  | 0.89 | 0.23 |  | 0.460^g^ | 0.000 |  | 0.93 | 0.21 |  | 0.90 | 0.23 |  | 0.82 | 0.23 |  | 0.000^f^ | 0.044 |
| Ethical concern | 0.90 | 0.15 |  | 0.89 | 0.15 |  | 0.092^g^ | 0.001 |  | 0.89 | 0.14 |  | 0.89 | 0.14 |  | 0.91 | 0.16 |  | 0.011^f^ | 0.003 |

^a^=ANOVA (equal variances, Levene's test p>0.05) or Welch test (unequal variances, Levene's test p<0.05) was used to test differences between genders and age groups.

^b^=Effect size was judged against criteria proposed by Cohen [32] for Partial Eta Squared: very small (<0.01), small (0.01 to <0.06), moderate (0.06 to <0.14), and large (≥ 0.14)

^c^=Beef, pork, lamb, game, offal, sausage and meat products

^d^=Bean, peas and soy products

^e^=Scores > 1 for each relative food motive reflect that it was rated more important compared to the mean and < 1 the opposite

^f^=Welch test

^g^=ANOVA

**Supplementary Table 2**. Mean consumption of red meat and legumes and relative food motive mean scores by BMI groups

|  | Participants without obesity | |  | Participants with obesity | |  | P^a^ | Partial Eta^2,b^ |
| --- | --- | --- | --- | --- | --- | --- | --- | --- |
|  | Mean | SD |  | Mean | SD |  |  |  |
| Red meat consumption^c^ (g/d) | 128 | 92.5 |  | 145 | 108.1 |  | 0.001^g^ | 0.004 |
| Legume consumption^d^ (g/d) | 16 | 16.1 |  | 17 | 19.6 |  | 0.187^g^ | 0.001 |
| Energy intake (kJ) | 9805 | 3637 |  | 9649 | 3709 |  | 0.348^g^ | 0.000 |
| Food motive^e^ |  | |  |  | |  |  |  |
| Health | 1.08 | 0.12 |  | 1.05 | 0.12 |  | 0.000^g^ | 0.015 |
| Mood | 0.98 | 0.15 |  | 0.99 | 0.15 |  | 0.154^g^ | 0.001 |
| Convenience | 0.98 | 0.21 |  | 1.02 | 0.23 |  | 0.000^g^ | 0.006 |
| Sensory appeal | 1.13 | 0.17 |  | 1.13 | 0.17 |  | 0.870^g^ | 0.000 |
| Natural Content | 1.02 | 0.20 |  | 0.98 | 0.21 |  | 0.000^g^ | 0.004 |
| Price-cheap | 1.01 | 0.25 |  | 1.06 | 0.26 |  | 0.000^g^ | 0.006 |
| Price-value | 1.18 | 0.21 |  | 1.19 | 0.20 |  | 0.710^g^ | 0.000 |
| Weight control | 0.96 | 0.18 |  | 0.98 | 0.16 |  | 0.003^f^ | 0.002 |
| Familiarity | 0.87 | 0.23 |  | 0.91 | 0.23 |  | 0.001^g^ | 0.003 |
| Ethical concern | 0.90 | 0.15 |  | 0.88 | 0.15 |  | 0.013^g^ | 0.002 |

^a^=ANOVA (equal variances, Levene's test p>0.05) or Welch test (unequal variances, Levene's test p<0.05) was used to test differences between genders and age groups.

^b^=Effect size was judged against criteria proposed by Cohen [32] for Partial Eta Squared: very small (<0.01), small (0.01 to <0.06), moderate (0.06 to <0.14), and large (≥0.14)

^c^=Beef, pork, lamb, game, offal, sausage and meat products

^d^=Bean, peas and soy products

^e^=Scores > 1 for each relative food motive reflect that it was rated more important compared to the mean and < 1 the opposite

^f^=Welch test

^g^=ANOVA
